# Clinicopathologic and genetic features of multiple system atrophy with Lewy body disease

**DOI:** 10.1111/bpa.12839

**Published:** 2020-04-14

**Authors:** Shunsuke Koga, Fuyao Li, Na Zhao, Shanu F. Roemer, Tanis J. Ferman, Anna I. Wernick, Ronald L. Walton, Ayman H. Faroqi, Neill R. Graff‐Radford, William P. Cheshire, Owen A. Ross, Dennis W. Dickson

**Affiliations:** ^1^ Department of Neuroscience Mayo Clinic Jacksonville FL; ^2^ Department of Psychiatry and Psychology Mayo Clinic Jacksonville FL; ^3^ Faculty of Biology, Medicine and Health University of Manchester Manchester UK; ^4^ Mayo Graduate School of Biomedical Sciences Mayo Clinic Jacksonville FL; ^5^ Department of Neurology Mayo Clinic Jacksonville FL

**Keywords:** *APOE*, dementia with Lewy bodies, *GBA*, minimal change MSA, multiple system atrophy

## Abstract

**Background:** Abnormal aggregates of α‐synuclein are pathologic hallmarks of multiple system atrophy (MSA) and Lewy body disease (LBD). LBD sometimes coexists with MSA, but the impact of co‐pathology, particularly diffuse LBD, on presentation of MSA has not been studied. We aimed to determine the frequency and clinicopathologic features of MSA with LBD (MSA+LBD). **Methods:** Using hematoxylin & eosin and α‐synuclein‐immunostained slides, we assessed the distribution and severity of LBD in 230 autopsy‐confirmed MSA patients collected from 1998 to 2018. Alzheimer‐type pathology was assessed to assign the likelihood of clinical presentations of dementia with Lewy body (DLB) using the consensus criteria for DLB. We reviewed medical records to characterize clinicopathologic features of MSA+LBD. Genetic risk factors for LBD, including *APOE* ε4 allele and mutations in *GBA*, *SNCA*, *LRRK2*, and *VPS35*, were analyzed. **Results:** LBD was observed in 11 MSA patients (5%); seven were brainstem type, three were transitional type, and one was diffuse type. The latter four had an intermediate or high likelihood of DLB. Three of the four had an antemortem diagnosis of Parkinson’s disease with dementia (PDD) or clinically probable DLB. Two patients had neuronal loss in the substantia nigra, but not in striatal or olivocerebellar systems with widespread glial cytoplasmic inclusions, consistent with minimal change MSA. In these cases, LBD was considered the primary pathology, and MSA was considered coincidental. *APOE* ε4 allele frequency was not different between MSA+LBD and MSA without LBD. Two of nine MSA+LBD patients had a risk variant of *GBA* (p.T408M and p.E365K). **Conclusions:** Although rare, MSA with transitional or diffuse LBD can develop clinical features of PDD or DLB. Minimal change MSA can be interpreted as a coincidental, but distinct, α‐synucleinopathy in a subset of patients with diffuse LBD.

## Introduction

Multiple system atrophy (MSA) and Lewy body disease (LBD) are neurodegenerative diseases pathologically characterized by neuronal and glial aggregates of α‐synuclein. Thus, they are collectively termed α‐synucleinopathies (33). In MSA, α‐synuclein forms aggregates in oligodendrocytes as glial cytoplasmic inclusions, but it also aggregates in a select subset of vulnerable neurons, especially those in the corpus striatum, pontine base, and inferior olivary nucleus (5, 36). Clinically, MSA is an atypical parkinsonian disorder characterized by a variable combination of autonomic failure, parkinsonism, cerebellar ataxia, and pyramidal signs (13, 43); REM sleep behavior disorder (RBD) is a frequent feature. In LBD, α‐synuclein forms aggregates mainly in neurons as Lewy bodies and Lewy neurites, but to a lesser extent in oligodendrocytes (40). LBD can be divided into three major subtypes based on the distribution of Lewy bodies: brainstem (BLBD), transitional (TLBD), and diffuse type (DLBD) (26). LBD can present with either Parkinson’s disease (PD), PD with dementia (PDD), or dementia with Lewy bodies (DLB) (6). PD is the most common neurodegenerative movement disorder characterized by bradykinesia, cogwheel rigidity, resting tremor, and postural instability (31). Autonomic dysfunction, such as constipation, is common in PD, and the majority of PD patients develop dementia in the later disease course, so‐called PDD (1, 31). DLB is the second most common neurodegenerative dementia and is characterized by two or more core features of fluctuating cognition, visual hallucinations, parkinsonism, or RBD. (25).

Although MSA and LBD are clinicopathologically distinct entities, clinical presentations of each disorder sometime overlap; thus, a subset of patients with LBD can be misdiagnosed as MSA (16, 27). Patients with MSA and DLB are both likely to have autonomic dysfunction (25) and RBD. Furthermore, 8%–23% of patients with MSA can have concomitant LBD (MSA+LBD) (14, 27, 30, 41). The majority of cases of LBD in MSA were considered to be BLBD or TLBD; the combination of DLBD and MSA has rarely been reported. The impact of concurrent LBD on clinical presentations in MSA has not been studied.

In this study, we aimed to determine the frequency of LBD in MSA, as well as its clinicopathologic and genetic features. To this end, we screened for LBD pathology in 230 autopsy‐confirmed MSA patients. They were also analyzed for genetic risk factors for LBD, including *APOE*, *GBA*, *SNCA*, *LRRK2*, and *VPS35*. We compared clinical and genetic features of MSA+LBD to MSA without LBD and to 652 LBD patients. We provide detailed clinicopathologic characteristics of MSA+LBD patients.

## Methods

### Subjects

All brains were from the Mayo Clinic brain bank and acquired between 1998 and 2018. This study included 230 consecutive cases of MSA (mean age: 66.8 ± 8.7 years) and 652 cases of LBD (mean age: 78.6 ± 8.1 years). We tabulated demographic, pathologic, and genetic features for the three groups: MSA, MSA+LBD, and LBD. The 652 LBD cases included in this study were previously reported (8). All brain autopsies were performed with the consent of the next‐of‐kin or an individual with legal authority to grant permission. De‐identified studies using these autopsy samples are considered exempt from human subject research by the Mayo Clinic Institutional Review Board.

### Neuropathological diagnosis of MSA

Immunohistochemistry for α‐synuclein (NACP, 1:3000 rabbit polyclonal, Mayo Clinic antibody, FL) (9) was performed on paraffin‐embedded 5‐μm thick sections of the basal forebrain, striatum, midbrain, pons, medulla, and cerebellum to establish a neuropathologic diagnosis of MSA (36). Following deparaffinization in xylene and reagent alcohol, antigen retrieval was performed by pretreatment with 95% formic acid for 30 minutes and then steam in distilled water for 30 minutes. All immunohistochemistry was conducted using a DAKO Autostainer and DAKO EnVision™ + reagents with 3,3′‐diaminobenzidine as the chromogen (Dako, Carpinteria, CA). Immunostained slides were counterstained with hematoxylin and coverslipped. MSA cases were pathologically divided into four types: predominant striatonigral involvement (SND), predominant olivopontocerebellar involvement (OPCA), equally severe involvement of striatonigral and olivopontocerebellar systems (SND+OPCA) (30), as well as minimal change MSA, which showed no or minimal neuronal loss in striatonigral or olivopontocerebellar systems despite widespread glial cytoplasmic inclusions (18, 20, 23, 42). To assess microglial activation, select cases were immunostained with an antibody to CD68 (1:1000, mouse monoclonal, DAKO, Carpinteria, CA). For the sections of substantia nigra, Mach‐2 Mouse‐AP Polymer Detection kit with Warp Red Chromogen was used.

### Neuropathological assessment of Lewy‐related pathology

**Figure 1:**
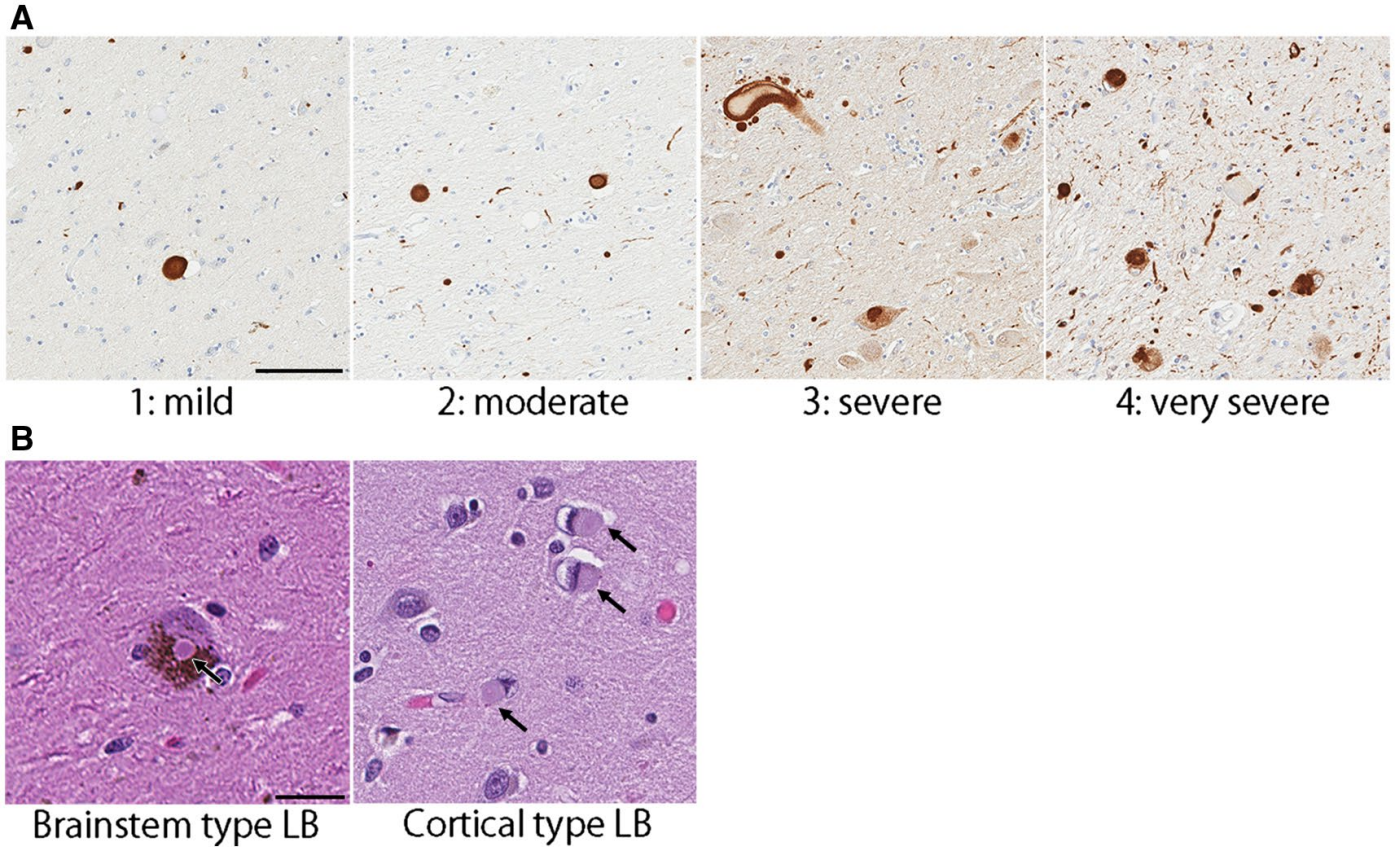
(**A**) Scoring of Lewy body disease pathology. Representative images of immunohistochemistry for α‐synuclein are taken from the nucleus basalis of Meynert. Bar = 100 μm. (**B**) Representative images of brainstem type and cortical type Lewy bodies (arrows) on hematoxylin & eosin stained slides. Bar = 30 μm.

Lewy‐related pathology was assessed in the neocortices (frontal, temporal, parietal, visual, and motor), cingulate gyrus, transentorhinal cortex, amygdala, basal forebrain, midbrain, pons, and medulla using hematoxylin & eosin (H&E) and α‐synuclein‐immunohistochemistry. In MSA+LBD cases, the severity of Lewy‐related pathology was graded semi‐quantitatively on a five‐point scale by two observers (S.K. and D.W.D.) based on the criteria of the third report of the DLB consortium (26) with some modifications as follows: score 0—absent; 1—sparse Lewy bodies or Lewy neurites; 2—≥2 Lewy body per high‐power (400×) field and sparse Lewy neurites; 3—≥4 Lewy bodies and scattered Lewy neurites in high‐power field; 4—numerous Lewy bodies and Lewy neurites. Figure 1A shows representative images of each score. Based on the distribution and severity of Lewy‐related pathology, they were classified as BLBD, TLBD, or DLBD. To distinguish Lewy bodies from neuronal cytoplasmic inclusions, H&E‐stained slides were also assessed; both brainstem and cortical type Lewy bodies were visible on H&E (Figure 1B), but neuronal cytoplasmic inclusions were not. Neuronal loss in vulnerable regions to LBD (ie, the dorsal motor nucleus of vagus nerve, locus coeruleus, and nucleus basalis of Meynert) was assessed semi‐quantitatively by two observers (S.K. and D.W.D.) on H&E‐stained slides: score 0—absent; 1—mild; 2—moderate; 3—severe neuronal loss (7).

### Neuropathological assessment of Alzheimer‐type pathology

All cases underwent a standardized neuropathological assessment for Alzheimer‐type pathology as previously described (22). Paraffin‐embedded 5‐μm thick sections from the neocortices (frontal, temporal, parietal, visual, and motor), hippocampus (CA1 and subiculum), entorhinal cortex, amygdala, basal ganglia, and cerebellum mounted on glass slides were assessed with thioflavin S fluorescent microscopy. Braak neurofibrillary tangle stages and Thal amyloid phases were assigned to each case based upon density and distribution of neurofibrillary tangles and senile plaques, respectively (3, 8, 35).

### Immunoblotting

For biochemical analysis of MSA+LBD cases, immunoblotting was performed using frozen brain samples from four patients with MSA+LBD (Cases 8–11), as well as four MSA, four DLBD, and two Alzheimer's disease cases (Table S1). Frozen brain tissues from superior temporal gyrus and cerebellum were homogenized (w/v) in 1X TBS (50 mM Tris, 150 mM NaCl, 1 mM PMSF, pH 7.6), supplemented with protease inhibitor (complete, Roche) and phosphatase inhibitor (PhosSTOP, Roche). The samples were centrifuged at 45 000 rpm for 60 minutes at 4°C in a Beckman TLA‐55 rotor (Beckman Coulter, Brea CA USA). The resulting supernatant was collected as TBS‐soluble fractions. The pellets were resuspended in TBS‐X (TBS with 1% Triton X‐100), supplemented with protease inhibitor and phosphatase inhibitor, and centrifuged at 45 000 rpm for 60 minutes at 4°C in a TLA‐55 rotor. The resulting supernatant was collected as TBS‐X‐soluble fractions. The pellets were resuspended in 2% sodium dodecyl sulfate (SDS) buffer and centrifuged at 45 000 rpm for 60 minutes at 4°C in a TLA‐55 rotor. The resulting supernatant was collected as SDS fractions.

An equal amount of protein from each fraction of the homogenized tissue lysates was resolved in sample buffer containing 2% SDS and 10% β‐mercaptoethanol. For immunoblotting, the samples were separated on 4%–20% Criterion Stain‐free gradient gels (Bio‐Rad, CA) (32). Gels were activated by UV exposure for 45 s using a Bio‐Rad Chemidoc MP imager to obtain images of total protein loading (32). After transferring to polyvinylidene difluoride membranes, the membranes were blocked with 5% skim milk in TBS‐T for 1 h and then incubated with anti‐α‐synuclein (phospho S129) antibody (EP1536Y, 1:2000, Abcam, Cambridge, MA) at 4°C overnight. Membranes were washed with TBS‐T three times for 5 minutes, incubated with goat anti‐rabbit horseradish peroxidase antibody (1:5000, Sigma, MO) at room temperature for 1 h, washed with TBS‐T three times for 10 minutes, and incubated with ECL chemiluminescence substrate (Bio‐Rad). Images were captured on the Chemidoc MP.

### Clinical Assessment

Clinical information was abstracted from available medical records or brain bank questionnaires that were filled out by a close family member (16). This included age of onset, age of death, sex, clinical diagnoses, family history of PD in first degree relatives, clinical symptoms, medications, neurological signs, results from autonomic reflex screening tests, imaging studies, cognitive screening measures, and neuropsychological assessments. When available, the Mini‐Mental State Examination (MMSE), Mattis Dementia Rating Scale (DRS), Montreal Cognitive Assessment (MoCA), or Kokmen Short Test of Mental Status (STMS) (19) scores were recorded. The following symptoms and neurological signs were abstracted from medical records: orthostatic hypotension, syncope, dizziness, urinary symptoms (eg, urinary urgency, urinary incontinence, and urinary retention), constipation, erectile dysfunction, hyposmia/anosmia, asymmetry of parkinsonism, resting tremor, bradykinesia, axial/limb rigidity, dystonia, postural instability, falls, gait ataxia, limb ataxia, nystagmus, pyramidal signs (eg, spasticity, hyperreflexia, and Babinski’s sign), dysphagia, cognitive impairment, fluctuations in cognition, depression, visual hallucinations, hypersomnia, and RBD (2, 13).

### Genetic analysis

Genetic mutations and variants that are considered risk factors for LBD were selected based on the effect size (medium‐to‐high and very high) and allele frequency (intermediate and rare), and included *APOE*, *GBA*, *SNCA*, *LRRK2*, and *VPS35* (8, 11, 21, 29, 37). Genomic DNA was extracted from the cerebellum of frozen brain tissue using standard procedures. Genotyping for *APOE* alleles (SNP rs429358 C/T and rs7412 C/T) was performed in 208 MSA cases with available frozen brain tissues. For MSA+LBD (N = 9; frozen brain tissues were unavailable in two cases), three common missense GBA mutations (p.E365K, p.T408M, p.N409S), SNCA p.A30P and p.A53T, LRRK2 p.G2019S, and VPS35 p.D620N were also assessed with TaqMan SNP genotyping assays (Applied Biosystems, Foster City, CA) as previously reported (28). Genotype calls were obtained with QuantStudio™ Real‐Time PCR Software (Applied Biosystems). The *APOE* data of 652 LBD cases were previously published (8).

Copy number variation of *SNCA* was assessed in the 9 MSA+LBD cases using TaqMan Copy Number Assay (Applied Biosystems, Foster City, CA) on the QuantStudio™ Flex7 Real‐Time PCR System. CEPH DNA (Centre d'Etude du Polymorphism Humain, Paris, France) was used as a reference control, and RNaseP was used for an endogenous control in each assay. Cases with known *SNCA* duplications or triplications were included as positive controls. Each sample was assayed in quadruplicate. Copy number assays were analyzed using QuantStudio™ Real‐Time PCR Software.

### Statistical analyses

All statistical analyses were performed using R 3.4.3 (The R Foundation for Statistical Computing, Vienna, Austria). Fisher’s exact test was performed for group comparisons of categorical data as appropriate. Analysis of variance (ANOVA) on ranks, followed by Steel‐Dwass post hoc test was used for analyses of continuous variables as appropriate. Multivariate logistic regression models were built to identify variables independently correlated with the presence of LBD pathology in MSA. P‐values <0.05 were considered statistically significant.

## Results

### Neuropathology

**Figure 2:**
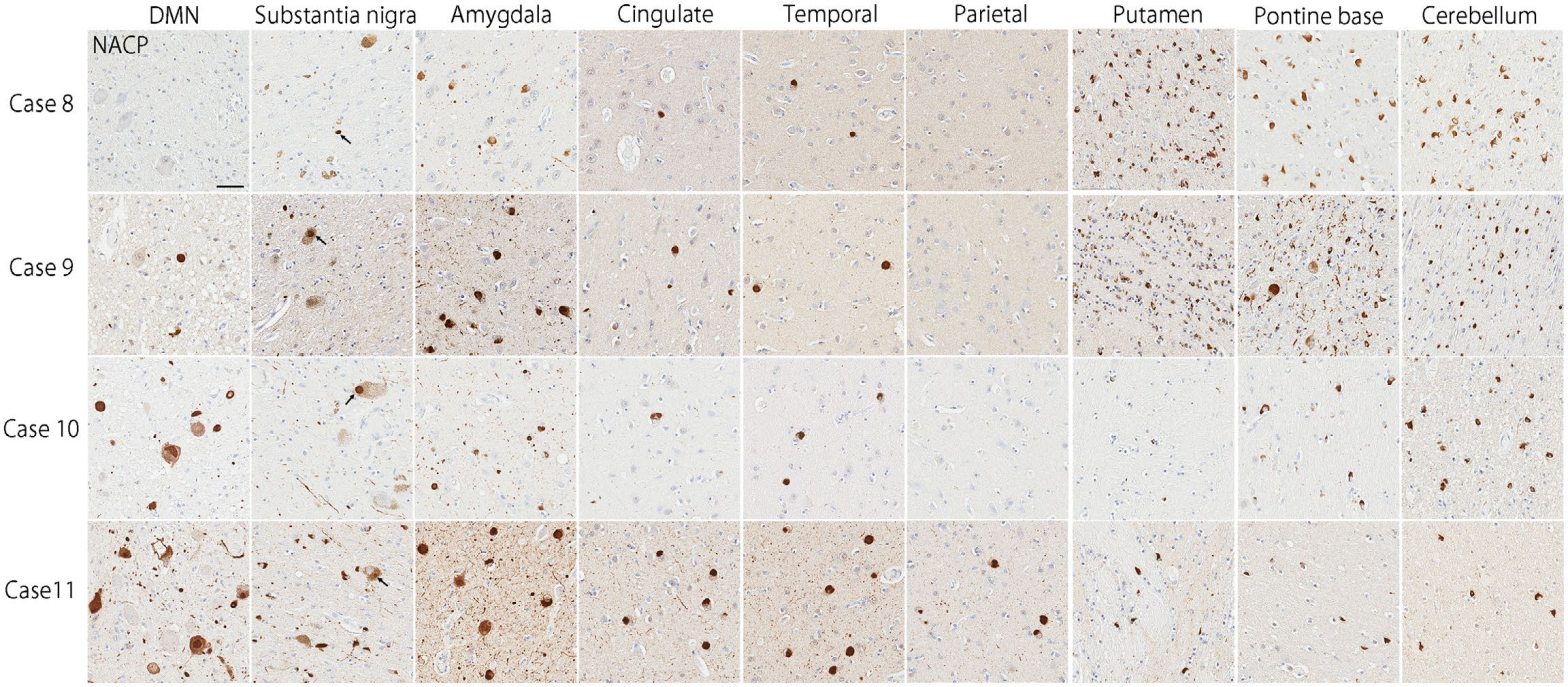
Representative images of immunohistochemistry for α‐synuclein (NACP) staining. Cases 8–10 show Lewy bodies mainly in the brainstem and the limbic structure, consistent with transitional type LBD. Case 11 shows abundant Lewy bodies in the brainstem, limbic structures, and neocortices, consistent with diffuse type LBD. Cases 8 and 9 have numerous glial cytoplasmic inclusions in the putamen, pontine base, and cerebellum, consistent with “typical” MSA. Neuronal cytoplasmic inclusions are also seen in the pontine base in Case 9. In contrast, glial cytoplasmic inclusions are observed, but much less than typical MSA cases, in Cases 10 and 11. Bar = 50 μm.

Of 230 autopsy‐confirmed MSA cases, 11 cases (5%) had concomitant LBD. The distribution and severity of Lewy bodies, neuronal loss in the select regions, subtypes of MSA, and Alzheimer‐type pathology are shown in Table 1. Seven cases (Case 1–7) had mild‐to‐moderate Lewy bodies in the brainstem, basal forebrain, and limbic structures, consistent with BLBD. Despite the presence of Lewy bodies, Case 1 did not have neuronal loss in the dorsal motor nucleus of the vagus nerve, locus coeruleus, and nucleus basalis of Meynert, consistent with incidental LBD (15). Three cases (Cases 8–10) had abundant Lewy bodies in the nucleus basalis of Meynert and amygdala, moderate in the transentorhinal cortex, and sparse Lewy bodies in the substantia nigra, frontal lobe, and temporal lobe, consistent with TLBD (Figure 2). One case (Case 11) had extensive Lewy bodies in the brainstem, basal forebrain and limbic regions, as well as neocortical regions, consistent with DLBD (Figure 2). Cases 10 and 11 had severe neuronal loss in the nucleus basalis of Meynert. Based on the LBD subtype and Alzheimer‐type pathology, the likelihood that the pathologic findings would be associated with DLB clinical syndrome was low in seven patients (Cases 1–7), intermediate in two patients (Cases 8 and 10), and high in two patients (Cases 9 and 11) as shown in Table 1 (25).

**Table 1:** Summary of pathologic and genetic features of MSA+LBD cases.

Case	Age	Sex	MSA subtype	LBD Type	Density of Lewy bodies										Neuronal loss			Braak	Thal	DLB Likelihood	APOE	GBA
					IX‐X	LC	SN	nbM	Amyg	Trans	Cing	Front	Temp	Par	DMN	LC	nbM					
1	73	M	SND	BLBD	1	0	1	1	0	0	0	0	0	0	0	0	0	I	0	Low	ε2ε3	–
2	79	F	SND	BLBD	3	1	1	1	0	0	0	0	0	0	2	2	0	I	1	Low	NA	NA
3	73	M	SND+OPCA	BLBD	1	2	2	2	1	0	0	0	0	0	1	2	0	II	3	Low	ε3ε4	–
4	68	M	SND+OPCA	BLBD	3	0	1	2	1	0	0	0	0	0	1	1	0	II	0	Low	ε3ε3	T408M
5	69	M	SND+OPCA	BLBD	3	3	1	2	2	0	0	0	0	0	0	2	1	II	3	Low	ε3ε3	–
6	76	M	SND	BLBD	2	3	2	2	2	1	0	0	0	0	2	2	0	IV	3	Low	NA	NA
7	73	M	OPCA	BLBD	1	3	2	3	3	1	0	0	0	0	0	2	0	II	0	Low	ε3ε3	–
8	68	F	SND	TLBD	0	0	1	3	4	2	0	1	1	0	1	2	0	IV	4	Inter	ε3ε3	–
9	70	M	SND+OPCA	TLBD	1	NA	1	4	4	3	1	1	1	0	0	NA	0	II	4	High	ε3ε4	–
10	75	M	Minimal	TLBD	3	3	3	2	2	2	2	1	1	0	2	1	3	III	3	Inter	ε3ε3	E365K
11	67	M	Minimal	DLBD	3	3	3	4	4	2	4	4	4	3	1	1	3	III	2	High	ε3ε4	–

Abbreviations: Amyg = amygdala; Braak = Braak neurofibrillary tangle stage; Cing = cingulate gyrus; DMN = dorsal motor nucleus of the vagus nerve; Front = frontal lobe; Inter = intermediate; IX‐X = 9th and 10th cranial nerve nucleus; LC = locus coeruleus; Minimal = minimal change MSA; nbM = nucleus basalis of Meynert; OPCA = olivopontocerebellar atrophy; Par = parietal lobe; SN = substantia nigra; SND = striatonigral degeneration; SND+OPCA = SND+OPCA; Temp = temporal lobe; Thal = Thal amyloid phase; Trans = Transentorhinal cortex.

**Figure 3:**
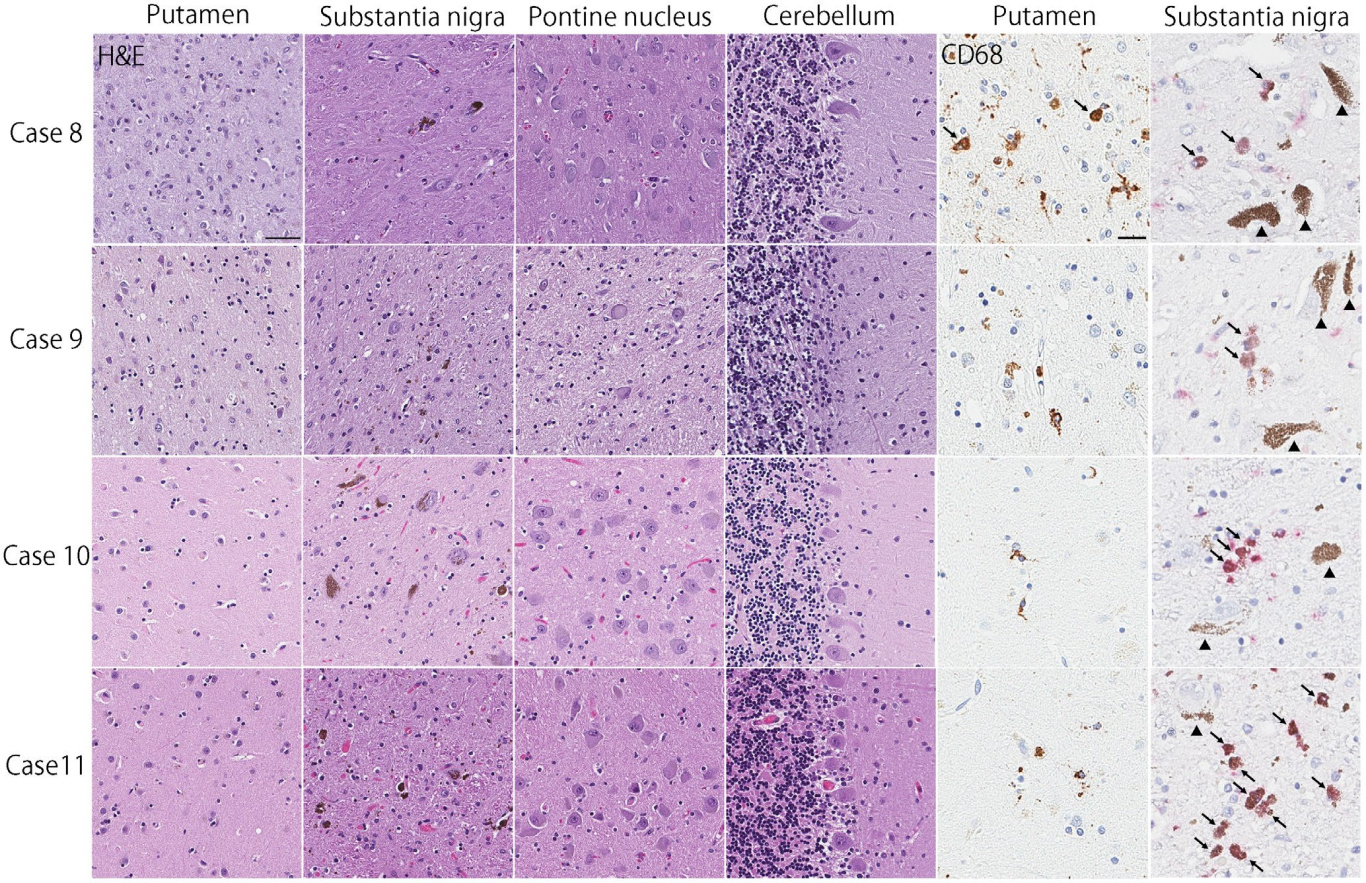
Hematoxylin & eosin staining shows severe neuronal loss and gliosis in the putamen and substantia nigra, as well as mild neuronal loss with gliosis in the cerebellum in Case 8. Severe neuronal loss and gliosis are observed in the putamen, substantia nigra, pontine nucleus, and cerebellum in Case 9. In Cases 10 and 11, neuronal and gliosis is restricted to the substantia nigra, consistent with “minimal change” MSA. Immunohistochemistry for CD68 shows amoeboid microglia (arrows) in the putamen in Case 8, but almost no amoeboid microglia in other three cases. In the substantia nigra, all cases have amoeboid microglia as shown in red chromogen (arrows). Arrowheads show neuromelanin pigments. Bars = 50 μm on Hematoxylin & eosin stained slides and 10 μm on CD68‐immunostained slides.

**Figure 4:**
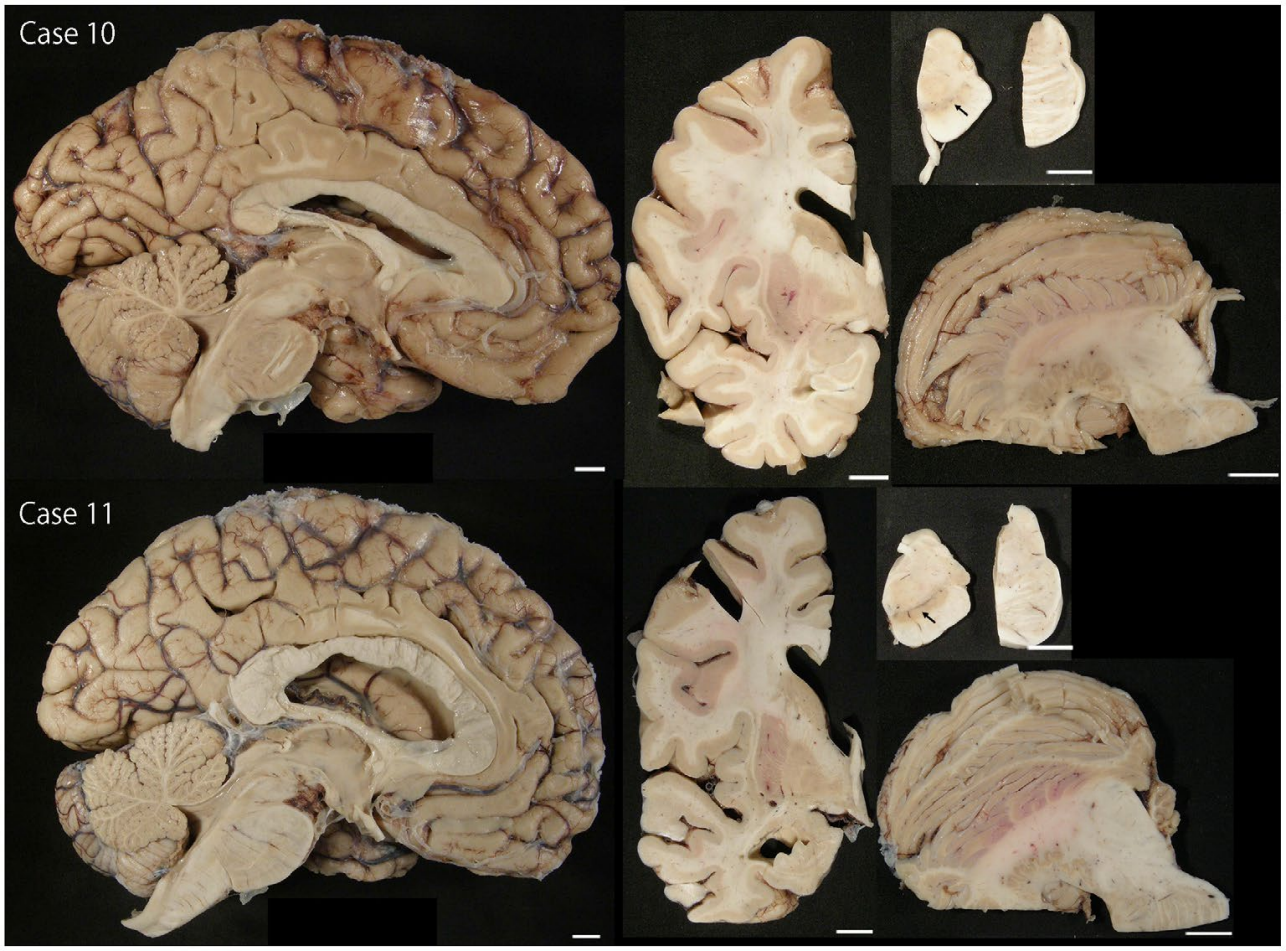
Macroscopic findings in Cases 10 and 11. The striatonigral and olivopontocerebellar structures are well preserved, except for the loss of pigment in the substantia nigra (arrows). Bars = 1 cm in all images.

Regarding the subtype of MSA, four cases had SND‐predominant, one case had OPCA‐predominant, and four cases had SND+OPCA type pathology (Table 1). The remaining two cases (Cases 10 and 11) had sparse, but widespread glial cytoplasmic inclusions throughout the brain, a few neuronal cytoplasmic inclusions, and dystrophic neurites in the pontine nuclei and inferior olivary nucleus (Figure 2). Neuronal loss and gliosis in these cases were restricted to the substantia nigra; they lacked significant neuronal loss and gliosis in cardinal brain regions, such as the putamen, pontine base, and cerebellum (Figure 3). This pathology is consistent with so‐called “minimal change MSA” (42). Macroscopically, loss of the pigment in the substantia nigra was noted, but atrophy of the cerebellum, brainstem, and putamen was not observed (Figure 4). Immunohistochemistry for CD68 also supported the diagnosis of minimal change MSA; activated microglia were present in the substantia nigra, particularly the ventrolateral part, but only a few ramified microglia were observed in the putamen (Figure 3).

Table 2 compares demographic and pathological features of MSA+LBD and MSA without LBD, as well as LBD patients. The age at death was oldest in LBD, followed by MSA+LBD and MSA. The age at death in MSA+LBD was significantly older than MSA without LBD (73 vs. 65 years, *P* = 0.012). The age at disease onset in MSA+LBD was nominally older than MSA without LBD (62 vs. 58 years, *P* = 0.07). The disease duration was not significantly different between the two groups (7 vs. 7 years, *P* = 0.81). The male/female ratio tended to be higher in MSA+LBD, but it did not reach statistical significance. The frequency of pathologic subtypes of MSA was different between the two groups. Minimal change MSA was more frequently observed in MSA+LBD than in MSA without LBD. As expected, Alzheimer‐type pathology was more severe, and *APOE* ε4 allele frequency was higher in LBD than the other two groups (8, 28). Comparison of MSA+LBD and MSA without LBD, using Steel‐Dwass post hoc test, demonstrated that the median scores of both Braak neurofibrillary tangle stage (II vs. I, *P* = 0.039) and Thal amyloid phase (3 vs. 0, *P* = 0.017) were higher in MSA+LBD than in MSA without LBD. A multivariate logistic regression model adjusting for age, Braak NFT stage, and Thal amyloid phase revealed that only Thal amyloid phase was associated with the presence of LBD pathology in MSA (OR: 1.78, 95% CI: 1.19–2.67, *P* = 0.005).

**Table 2:** Demographic, pathologic, and genetic features of 230 MSA and 652 LBD patients.

	MSA N = 219	MSA+LBD N = 11	LBD N = 652	*P* value
%Male (N)	53% (116)	82% (9)	58% (381)	0.098
Age at death, years	65 (60, 71)	73 (68, 74)	79 (74, 84)	<0.001
Age at onset, years	58 (53, 64)	62 (60, 67)	66 (71, 77)	<0.001
Disease duration, years	7 (5, 9)	7 (6, 8)	8 (5, 11)	<0.001
Brain weight, grams	1220 (1120, 1320)	1280 (1210, 1340)	1140 (1040, 1260)	<0.001
Pathologic subtype				
SND predominant	44% (97)	36% (4)	–	0.014
OPCA predominant	18% (39)	9% (1)	–	
SND+OPCA	37% (82)	36% (4)	–	
Minimal change	1% (1)	18% (2)	–	
LBD subtype				
Brainstem LBD	–	64% (7)	12% (75)	<0.001
Transitional LBD	–	27% (3)	34% (219)	
Diffuse LBD	–	9% (1)	55% (358)	
Alzheimer‐type pathology				
Braak neurofibrillary tangle stage	I (I, II)	II (II, III)	IV (III, V)	<0.001
Thal Aβ phase	0 (0, 2)	3 (0, 3)	4 (3, 5)	<0.001
*APOE* alleles	N = 199	N = 9	N = 652	
ε2 allele frequency	9% (36/398)	6% (1/18)	4% (58/1304)	<0.001
ε3 allele frequency	77% (308/398)	78% (14/18)	64% (831/1304)	
ε4 allele frequency	14% (54/398)	17% (3/18)	32% (415/1304)	

Data are % (n) or median (25th, 75th %‐tile).

Abbreviations: LBD = Lewy body disease; MSA = multiple system atrophy; OPCA = olivopontocerebellar atrophy; SND = striatonigral degeneration.

### Biochemical analysis

**Figure 5:**
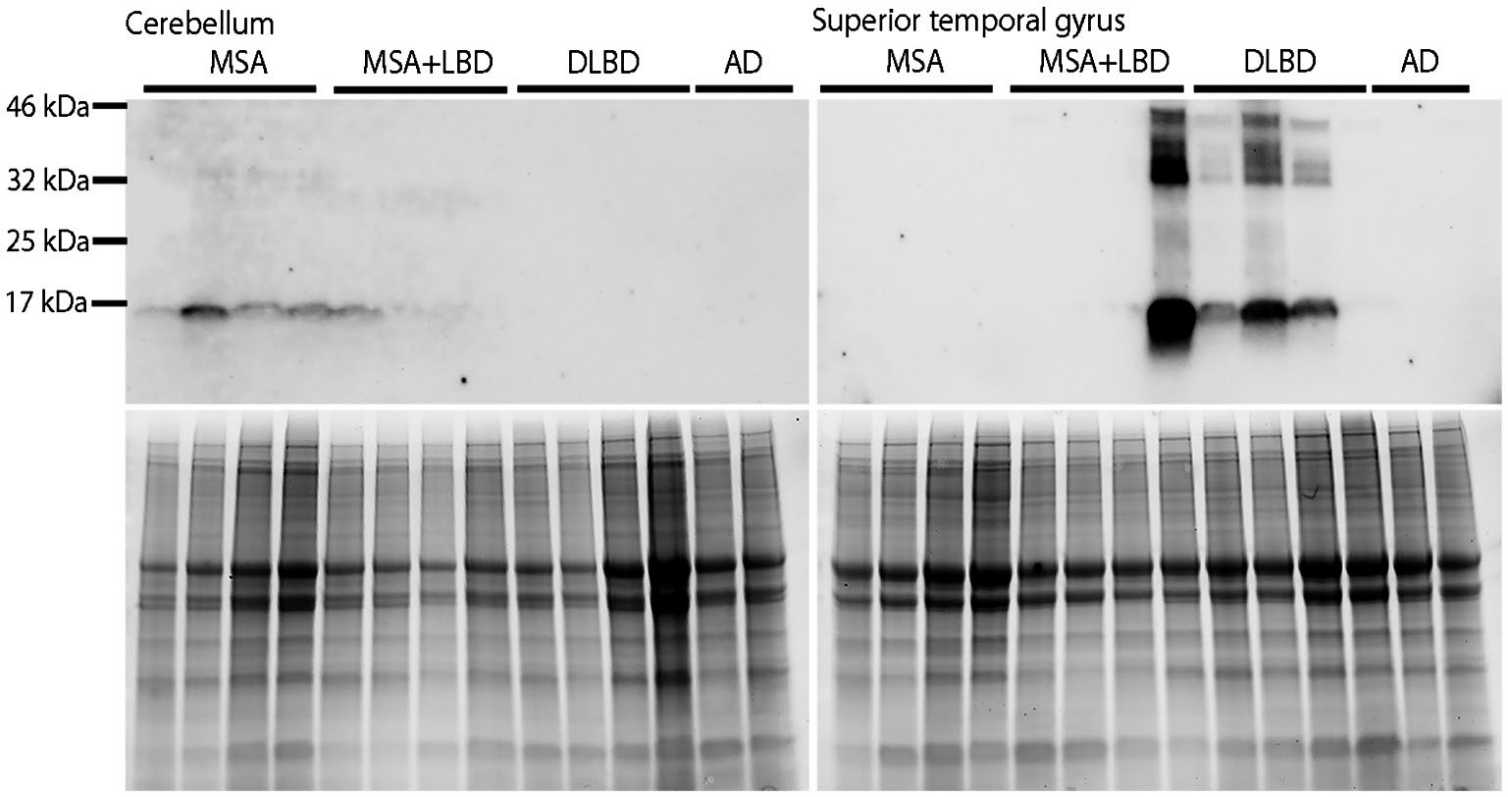
Immunoblotting of phosphorylated‐α‐synuclein (upper panels) in MSA (n = 4), MSA+LBD (n = 4; Case 8–11), DLBD (n = 4), and Alzheimer's disease (n = 2) using SDS fractions from the cerebellum (left) and superior temporal gyrus (right). Total protein is shown as loading control (lower panels). Demographic information of MSA, DLBD, and Alzheimer's disease patients are shown in Table S1.

To determine the predominant molecular phenotype of α‐synuclein in MSA+LBD, we performed immunoblot analysis using SDS fractions from the superior temporal gyrus and cerebellum of Case 8–11. Case 11 had prominent phosphorylated‐α‐synuclein immunoreactive bands at approximately 17 kDa, which was similar to that observed in the superior temporal cortex in DLBD. In contrast, Case 11 did not have phosphorylated‐α‐synuclein immunoreactive bands in the cerebellum, as observed in MSA (Figure 5). This finding indicates that DLBD is the primary pathology, and MSA is coincidental in Case 11. Phosphorylated‐α‐synuclein immunoreactive bands were not detected in samples of TBS‐fractions and TBS‐X fractions (Figure S1).

### Clinical features of MSA+LBD

Table 3 summarizes clinical features of 11 MSA+LBD cases. Seven patients had a clinical diagnosis of MSA, two patients were diagnosed with DLB, one was PDD, and one was PSP. We provide a brief clinical vignette of each patient except for Case 2 whose medical records were unavailable for review.

**Table 3:** Clinical features of MSA with LBD.

Case	1	2	3	4	5	6	7	8	9	10	11
Clinical diagnosis	PSP	MSA	MSA‐P	MSA‐P	MSA‐P	MSA‐P	MSA‐C	DLB	MSA‐C	DLB	PDD
Family history of PD	NA	−	−	−	−	+	−	−	−	+	NA
Age at death	73	79	73	68	69	76	73	68	70	75	67
Age at onset	<57	NA	67	60	62	71	67	62	58	68	58
Disease duration	>6	NA	6	8	7	5	6	6	12	7	9
Initial symptoms	NA	NA	Unsteady gait	Bradykinesia	Bradykinesia	Shuffling gait	Imbalance, falls	Urinary incontinence	Ataxic gait	Cognitive impairment	Resting tremor
Parkinsonism	+	NA	+	+	+	+	+	+	+	+	+
Autonomic failure	+	NA	+	+	+	+	+	+	+	+	+
Ataxia	−	NA	+	+	−	−	+	−	+	−	−
Dysphagia	+	NA	−	+	+	+	+	+	−	+	+
Cognitive impairment	−	NA	−	−	−	+	+	+	−	+	+
Fluctuating cognition	−	NA	−	−	−	−	−	+	−	+	+
Visual hallucinations	−	NA	NA	−	+*	−	−	+	NA	+	+
RBD	+	NA	+	+	+	−	−	+	−	+	+
Hypersomnia	+	NA	NA	NA	NA	NA	+	NA	−	+	−
Depression	−	NA	NA	+	+	+	NA	+	−	−	+

Medical records are not available for review in Case 2.

* Visual hallucinations are considered to be caused by atropine.

Abbreviations: DLB = dementia with Lewy bodies; MSA = multiple system atrophy; MSA‐C = MSA‐cerebellar type; MSA‐P = MSA‐parkinsonism type; NA = not available; PD = Parkinson’s; PDD = Parkinson’s disease with dementia; PSP = Progressive supranuclear palsy; RBD = rapid eye movement sleep behavioral disorder.

#### Case 1

This 73‐year‐old Caucasian man had at least a 6‐year history of PD with wearing‐off phenomena and RBD. He subsequently developed frequent backward falls, blurry vision, diplopia, neck rigidity, urinary incontinence, stridor, dysphagia, and dysarthria at age 71. His vertical saccades were slow, and he had limited upgaze. He did not have cognitive impairment or visual hallucinations. His final clinical diagnosis was PSP.

#### Case 3

This 73‐year‐old Caucasian man presented with an unsteady gait, slurred speech, and difficulty handwriting at age 67. He also developed constipation, urinary incontinence, and erectile dysfunction. At age 68, neurologic examination revealed right‐predominant parkinsonism and ataxic dysarthria. An autonomic reflex study confirmed orthostatic hypotension and sudomotor dysfunction. A sleep study confirmed RBD. Based on these findings, he was clinically diagnosed with MSA.

#### Case 4

This 68‐year‐old Caucasian man presented with bradykinesia, unsteady gait, and difficulty with handwriting at age 60. His initial diagnosis was PD. He subsequently developed erectile dysfunction, urinary urgency and incontinence, and constipation at age 61. He also had RBD but did not have cognitive impairment or visual hallucinations. Diagnosis of MSA was made at age 64 due to levodopa‐unresponsive parkinsonism and early‐onset autonomic dysfunction.

#### Case 5

This 69‐year‐old Caucasian man had a 7‐year history of parkinsonism, including bradykinesia, dysarthria, difficulty with handwriting, and hypomimia. His initial diagnosis was PD. He did not have a good response to carbidopa/levodopa over the years. At age 68, he developed urinary retention, orthostatic hypotension, and stridor while sleeping. His cognition was intact. He had episodes of visual hallucinations after taking atropine for sialorrhea. Based on his levodopa‐unresponsiveness parkinsonism and autonomic dysfunction, he was diagnosed with MSA.

#### Case 6

This 76‐year‐old Caucasian man, with a family history of PD in his mother, developed shuffling gait at age 71. Neurological examination at age 73 revealed hypomimia, bradykinesia, bilateral rigidity, mildly kyphotic posture, and slow gait with reduced arm swing. Neither tremor nor dyskinesia was noted. He showed processing speed and memory difficulties with a MoCA score of 25/30. He also had urinary retention, orthostatic hypotension, erectile dysfunction, and stridor. He did not respond Carbidopa/levodopa. He was diagnosed with MSA due to levodopa‐unresponsive parkinsonism and autonomic dysfunction.

#### Case 7

This 73‐year‐old Caucasian man presented with a progressive decline in his balance with falls at age 67. He developed slurring speech and difficulty swallowing, as well as orthostatic hypotension at age 68. Neurological examination at age 70 revealed dysmetric saccades with intermittent gaze‐evoked nystagmus, limb ataxia with finger‐to‐nose and heel‐to‐shin, mild left‐sided rigidity, bradykinesia and rest tremor in the left upper extremity, as well as ataxic gait and retropulsion. He had mild cognitive deficits in memory, attention, and visuospatial function (STMS = 31/38). Because of the prominent cerebellar features with parkinsonism and autonomic dysfunction, he was diagnosed with MSA‐cerebellar type (MSA‐C).

#### Case 8

This 68‐year‐old Caucasian woman developed urinary incontinence at age 63, levodopa‐responsive parkinsonism, and polysomnographic‐confirmed RBD at age 64, as well as fully formed visual hallucinations and fluctuating cognition at age 65. At age 66, neurologic examination revealed square wave jerks, slow vertical saccades, rigidity (axial > limb), enhanced jaw jerk, mild stooped posture, anterocollis, a wide‐based and short‐stride gait with decreased arm swing, postural instability, and mixed dysarthria with spastic and hypokinetic components. She also had orthostatic hypotension and the “cold hands” sign. Based on symmetric parkinsonism without resting tremor, autonomic dysfunction, and RBD, she was diagnosed with probable MSA. Neuropsychological testing conducted at age 67 revealed mild‐to‐moderate dementia (DRS = 104/144), with a cognitive pattern of moderately slowed processing speed, heightened distractibility on tasks of divided attention, and impaired visuospatial skills, as well as low average memory and naming performance. MRI of the brain obtained at age 67 was unremarkable. She had no family history of known neurodegenerative disease. Based on these findings, her final clinical diagnosis was DLB.

#### Case 9

This 70‐year‐old Caucasian man developed imbalance with falls at age 58, and problems with urinary urgency and incontinence at age 59. At age 63, he was evaluated by a movement disorder specialist and found to have gait ataxia (Scale for the Assessment and Rating of Ataxia = 12), mild limb ataxia, and slight ataxic dysarthria, but no bradykinesia, rigidity or pyramidal signs. He retrospectively reported that he had problems with urinary control four years before. MRI of the brain, neck, cervical spine, and lumbar spine were unremarkable. Genetic testing for SCA1, SCA2, SCA3, SCA6, SCA7, SCA8, SCA10, SCA14, and SCA17, as well as dentato‐rubro‐pallido‐Luysial atrophy and Friedreich's ataxia, were all negative. Other acquired causes of ataxia were also excluded, including hypovitaminosis E, and autoantibodies to GAD and gliadin. Cognition was reportedly normal, but no formal neuropsychologic testing was carried out. Examination at age 68 revealed mild bradykinesia and rigidity in the extremities, limb ataxia, ataxic gait, and ataxic dysarthria. Based on the presence of progressive cerebellar ataxia and features of autonomic dysfunction, he was diagnosed with MSA‐C

#### Case 10

This 75‐year‐old Caucasian man had a long history of anosmia since his 20s and clinically probable RBD that started at age 50. At age 68, he developed cognitive difficulties with fluctuations that responded well to donepezil. At age 68, he developed stooped posture, hypomimia, sialorrhea, micrographia, and gait difficulty. Neurological examination revealed saccadic intrusions in smooth pursuits, significantly restricted upward gaze without nystagmus, and rigidity in upper extremities. At age 70, neuropsychological evaluation was consistent with non‐amnestic mild cognitive impairment and characterized by attention and visuospatial deficits (MMSE = 26/30, DRS = 132/144). MRI of the brain showed minimal small vessel disease, but was otherwise normal. At age 70, neurological examination revealed rigidity, a shuffling gait with decreased arm swing, and postural instability. Carbidopa‐levodopa was initiated at age 72 with good response. He developed orthostatic hypotension, urinary incontinence, and heat intolerance confirmed with an autonomic reflex study at age 72. Visual misperceptions were apparent at age 70, and fully formed visual hallucinations began at age 73. A repeat neurocognitive evaluation at age 74 continued to show non‐amnestic mild cognitive impairment, with disproportionate attention and visual perceptual deficits. Based on these findings, her final clinical diagnosis was DLB.

#### Case 11

This 67‐year‐old Caucasian man developed bilateral resting tremor at age 58. At age 64, neurologic examination revealed mild bilateral bradykinesia, mild resting tremor (worse on the right), and gait instability. He responded to carbidopa‐levodopa. MRI of the brain was unremarkable. At age 66, he developed probable RBD, fully formed visual hallucinations, and cognitive difficulties (MoCA = 23/30). At that time, he also exhibited dyskinesia, restless leg syndrome, urinary frequency, and constipation. He was clinically diagnosed with PDD and had a positive response to donepezil.

### Genetic analysis

To clarify whether MSA+LBD patients had genetic risk factors for LBD (21, 29), we screened mutations in SNCA (p.A30P and p.A53T), LRRK2 (p.G2019S), and VPS35 (p.D620N), as well as copy number of SNCA; however, all patients had a wild type and had normal copy number of *SNCA*. We also screened risk variants of *GBA* and found that two patients with MSA+LBD had the variants: p.T408M in Case 4 and p.E365K in Case 10.

## Discussion

Of 230 MSA cases, we found that 11 (5%) had concurrent LBD. This frequency is lower than those reported by others, but similar to a recent and extensive study by Miki *et al* (8%; 12/160) (14, 27, 30, 41). Concomitant LBD, without significant neuronal loss, has been observed in cognitively and neurologically normal elderly individuals, referred to as incidental LBD (7%–24%) (4, 12, 24). Concomitant LBD is reported in similar frequency in neurodegenerative diseases, such as PSP (11%) (38). In the present study, the main LBD subtype was BLBD. In Case 1 LBD was considered incidental LBD because of the lack of neuronal loss in the brain regions vulnerable to LBD. Clinical presentations of these patients (Case 1–7) seemed to be consistent with MSA, and six of them were clinically diagnosed with MSA. Although it is rare, MSA with coexisting TLBD or DLBD can present with dementia and visual hallucinations. Interestingly, two of these patients lacked significant neurodegeneration in striatonigral and olivopontocerebellar systems, consistent with minimal change MSA. In such cases, LBD is considered the primary pathologic process, and MSA is considered a coincidental pathology. Immunoblotting also confirmed that phosphorylated‐α‐synuclein immunoreactive bands of Case 11 resembled those observed in DLBD rather than MSA.

Given that clinical features of MSA and LBD overlap, it is challenging to assess the clinical impact of LBD pathology in MSA. Although there is a controversy regarding cognitive impairment in MSA (17, 34), dementia is a prerequisite feature for PDD and DLB (13). Indeed, three of four patients with an intermediate‐ or high‐likelihood of DLB presented with dementia and visual hallucinations, which is characteristic of PDD and DLB. Furthermore, clinical features addressed in research on prodromal LBD (2) were present in MSA+LBD, namely, depression (5/11) and hypersomnia (3/11). Nevertheless, we consider MSA the primary pathology and TLBD secondary in Cases 8 and 9. Case 8 was initially diagnosed with probable MSA at age 62, and symptoms suggestive of LBD did not emerge until age 67. Case 9 was clinically diagnosed with MSA‐C at age 63 due to the presence of cerebellar ataxia, urinary problems, and mild parkinsonism in the absence of dementia or visual hallucinations.

In contrast, the primary diagnosis of Cases 10 and 11 is considered to be LBD because neurodegeneration in the striatonigral and olivopontocerebellar structures was minimal. In addition, both cases had severe neuronal loss in the nucleus basalis of Meynert, where is usually does not have significant neuronal loss in MSA. Neither patient had cerebellar ataxia or other red flags for MSA (13); therefore, all their symptoms (ie, parkinsonism, autonomic dysfunction, dementia, visual hallucinations, and RBD) could be explained by LBD. Although it is challenging to determine the role MSA pathology might have had, most of the clinical features can be readily explained by Lewy‐related pathology. In these cases, minimal MSA pathology can be considered incidental. Incidental MSA was rare in the 652 LBD cases studied—a frequency of 0.3% (2/654). Interestingly, this frequency is similar to that of incidental MSA in PSP (0.3%; 1/290) (39) and in neurologically normal individuals (0.8%; 1/125) (10).

The unusual combination of MSA and DLBD encouraged us to analyze genetic risk factors. Although we did not identify any Mendelian forms of disease, five of nine MSA+LBD patients had either *APOE* ε4 allele or *GBA* risk variant. Given the fact that MSA+LBD patients had more severe Alzheimer‐type pathology than MSA without LBD, LBD pathology in MSA had the same genetic risk factors seen in LBD. In addition, the multivariate logistic regression model indicated that Thal amyloid phase was associated with LBD pathology in MSA. This indicates that LBD pathology occurs and progresses independently from MSA pathology in MSA+LBD patients.

A limitation of the study was that clinical information was from retrospective chart review and not from a systematic standardized clinical study. Symptoms suggestive of LBD, such as dementia, psychiatric symptoms, and RBD may have been missed in some patients. The strengths of our study are the number of MSA cases screened and the stringent methods used to distinguish Lewy bodies from MSA neuronal cytoplasmic inclusions. This might be a reason for the relatively lower frequency of concomitant LBD in MSA in our study compared to others, since other studies may have considered neuronal cytoplasmic inclusions as Lewy bodies (14, 27, 30, 41).

In conclusion, 5% of MSA patients had concomitant LBD. MSA patients with coexisting TLBD and DLBD can develop dementia and visual hallucinations, and in those cases, a clinical diagnosis of PDD or DLB may be more likely. Although parkinsonism, autonomic dysfunction, and RBD can be seen in both MSA and LBD, the presence of dementia and visual hallucinations, or dementia with RBD or parkinsonism should be considered as red flags for coexisting Lewy‐related pathology.

## Conflict of Interest

Dr. Koga receives support from a Karin & Sten Mortstedt CBD Solutions research grant. Ms. Li reports no disclosures. Dr. Zhao reports no disclosures. Dr. Roemer reports no disclosures. Dr. Ferman is supported by the Mangurian Foundation for Lewy body disease research and NIH. Ms. Wernick reports no disclosures. Mr. Walton reports no disclosures. Mr. Faroqi reports no disclosures. Dr. Graff‐Radford reports no disclosures. Dr. Cheshire is a consultant for American Academy of Neurology, Neuro SAE examination writer, 2013; and receives support from NIH, Autonomic Rare Diseases Clinical Research Consortium. He is an editorial board member of Autonomic Neuroscience. Dr. Ross receives support from R01‐NS078086, P50‐NS072187, U54 NS110435 and U54 NS100693, The Little Family Foundation, the Michael J. Fox Foundation, Mayo Clinic Center for Individualized Medicine, and The Functional Genomics of LBD Program at the Mayo Clinic. O.A.R. is an editorial board member of American Journal of Neurodegenerative Disease, Frontiers Neurology: Neurogenetics, and Molecular Neurodegeneration. Dr. Dickson receives support from the NIH (P01 AG003949, P30 AG062677, U01 NS100620, UG3 NS104095, RF1 AG057181). Dr. Dickson is an editorial board member of Acta Neuropathologica, Annals of Neurology, Brain, Brain Pathology, and Neuropathology, and he is editor in chief of American Journal of Neurodegenerative Disease, and International Journal of Clinical and Experimental Pathology.

## Supporting information


**Figure S1.** Immunoblotting of phosphorylated‐α‐synuclein (upper panels) in MSA (n = 4), MSA+LBD (n = 4), DLBD (n = 4), and Alzheimer's disease (n = 2) using TBS and TBS‐X fractions from the cerebellum (left) and superior temporal gyrus (right). Total protein is shown as loading control (lower panels). Phosphorylated‐α‐synuclein bands are not detected in all samples in TBS and TBS‐X fractions.
**Table S1.** Characteristics of cases for immunoblotting.

## Data Availability

The data that support the findings of this study are available from the corresponding author upon request.

## References

[1] Aarsland D , Kurz MW (2010) The epidemiology of dementia associated with Parkinson disease. J Neurol Sci 289:18–22. 19733364 10.1016/j.jns.2009.08.034

[2] Berg D , Postuma RB , Adler CH , Bloem BR , Chan P , Dubois B *et al* (2015) MDS research criteria for prodromal Parkinson's disease. Mov Disord 30:1600–1611. 26474317 10.1002/mds.26431

[3] Braak H , Braak E (1991) Neuropathological stageing of Alzheimer‐related changes. Acta Neuropathol 82:239–259. 1759558 10.1007/BF00308809

[4] Braak H , Del Tredici K , Rub U , de Vos RA , Jansen Steur EN , Braak E (2003) Staging of brain pathology related to sporadic Parkinson's disease. Neurobiol Aging 24:197–211. 12498954 10.1016/s0197-4580(02)00065-9

[5] Cykowski MD , Coon EA , Powell SZ , Jenkins SM , Benarroch EE , Low PA *et al* (2015) Expanding the spectrum of neuronal pathology in multiple system atrophy. Brain 138(Pt 8):2293–2309. 25981961 10.1093/brain/awv114 PMC4840945

[6] Dickson DW (2018) Neuropathology of Parkinson disease. Parkinsonism Relat Disord 46(Suppl. 1):S30–S33. 28780180 10.1016/j.parkreldis.2017.07.033 PMC5718208

[7] Dickson DW , Braak H , Duda JE , Duyckaerts C , Gasser T , Halliday GM *et al* (2009) Neuropathological assessment of Parkinson's disease: refining the diagnostic criteria. Lancet Neurol 8:1150–1157. 19909913 10.1016/S1474-4422(09)70238-8

[8] Dickson DW , Heckman MG , Murray ME , Soto AI , Walton RL , Diehl NN *et al* (2018) APOE epsilon4 is associated with severity of Lewy body pathology independent of Alzheimer pathology. Neurology 91:e1182–e1195. 30143564 10.1212/WNL.0000000000006212 PMC6161556

[9] Dickson DW , Liu W , Hardy J , Farrer M , Mehta N , Uitti R *et al* (1999) Widespread alterations of alpha‐synuclein in multiple system atrophy. Am J Pathol 155:1241–1251. 10514406 10.1016/s0002-9440(10)65226-1 PMC1867032

[10] Fujishiro H , Ahn TB , Frigerio R , DelleDonne A , Josephs KA , Parisi JE *et al* (2008) Glial cytoplasmic inclusions in neurologically normal elderly: prodromal multiple system atrophy? Acta Neuropathol 116:269–275. 18553090 10.1007/s00401-008-0398-7 PMC2880173

[11] Gasser T (2015) Usefulness of genetic testing in PD and PD trials: a balanced review. J Parkinsons Dis 5:209–215. 25624421 10.3233/JPD-140507 PMC4923738

[12] Gibb WR , Lees AJ (1988) The relevance of the Lewy body to the pathogenesis of idiopathic Parkinson's disease. J Neurol Neurosurg Psychiatry 51:745–752. 2841426 10.1136/jnnp.51.6.745 PMC1033142

[13] Gilman S , Wenning GK , Low PA , Brooks DJ , Mathias CJ , Trojanowski JQ *et al* (2008) Second consensus statement on the diagnosis of multiple system atrophy. Neurology 71:670–676. 18725592 10.1212/01.wnl.0000324625.00404.15 PMC2676993

[14] Jellinger KA (2007) More frequent Lewy bodies but less frequent Alzheimer‐type lesions in multiple system atrophy as compared to age‐matched control brains. Acta Neuropathol 114:299–303. 17476513 10.1007/s00401-007-0227-4

[15] Klos KJ , Ahlskog JE , Josephs KA , Apaydin H , Parisi JE , Boeve BF *et al* (2006) Alpha‐synuclein pathology in the spinal cords of neurologically asymptomatic aged individuals. Neurology 66:1100–1102. 16606927 10.1212/01.wnl.0000204179.88955.fa

[16] Koga S , Aoki N , Uitti RJ , van Gerpen JA , Cheshire WP , Josephs KA *et al* (2015) When DLB, PD, and PSP masquerade as MSA: an autopsy study of 134 patients. Neurology 85:404–412. 26138942 10.1212/WNL.0000000000001807 PMC4534078

[17] Koga S , Dickson DW (2018) Recent advances in neuropathology, biomarkers and therapeutic approach of multiple system atrophy. J Neurol Neurosurg Psychiatry 89:175–184. 28860330 10.1136/jnnp-2017-315813

[18] Koga S , Dickson DW (2019) “Minimal change” multiple system atrophy with limbic‐predominant alpha‐synuclein pathology. Acta Neuropathol 137:167–169. 30128820 10.1007/s00401-018-1901-4 PMC6339590

[19] Kokmen E , Naessens JM , Offord KP (1987) A short test of mental status: description and preliminary results. Mayo Clin Proc 62:281–288. 3561043 10.1016/s0025-6196(12)61905-3

[20] Kon T , Mori F , Tanji K , Miki Y , Wakabayashi K (2013) An autopsy case of preclinical multiple system atrophy (MSA‐C). Neuropathology 33:667–672. 23581648 10.1111/neup.12037

[21] Kumar KR , Weissbach A , Heldmann M , Kasten M , Tunc S , Sue CM *et al* (2012) Frequency of the D620N mutation in VPS35 in Parkinson disease. Arch Neurol 69:1360–1364. 22801713 10.1001/archneurol.2011.3367

[22] Liesinger AM , Graff‐Radford NR , Duara R , Carter RE , Hanna Al‐Shaikh FS , Koga S *et al* (2018) Sex and age interact to determine clinicopathologic differences in Alzheimer's disease. Acta Neuropathol 136:873–885. 30219939 10.1007/s00401-018-1908-x PMC6280837

[23] Ling H , Asi YT , Petrovic IN , Ahmed Z , Prashanth LK , Hazrati LN *et al* (2015) Minimal change multiple system atrophy: an aggressive variant? Mov Disord 30:960–967. 25854893 10.1002/mds.26220

[24] Markesbery WR , Jicha GA , Liu H , Schmitt FA (2009) Lewy body pathology in normal elderly subjects. J Neuropathol Exp Neurol 68:816–822. 19535990 10.1097/NEN.0b013e3181ac10a7 PMC2704264

[25] McKeith IG , Boeve BF , Dickson DW , Halliday G , Taylor JP , Weintraub D *et al* (2017) Diagnosis and management of dementia with Lewy bodies: fourth consensus report of the DLB Consortium. Neurology 89:88–100. 28592453 10.1212/WNL.0000000000004058 PMC5496518

[26] McKeith IG , Dickson DW , Lowe J , Emre M , O'Brien JT , Feldman H *et al* (2005) Diagnosis and management of dementia with Lewy bodies: third report of the DLB Consortium. Neurology 65:1863–1872. 16237129 10.1212/01.wnl.0000187889.17253.b1

[27] Miki Y , Foti SC , Asi YT , Tsushima E , Quinn N , Ling H , Holton JL (2019) Improving diagnostic accuracy of multiple system atrophy: a clinicopathological study. Brain 142:2813–2827. 31289815 10.1093/brain/awz189

[28] Ogaki K , Martens YA , Heckman MG , Koga S , Labbe C , Lorenzo‐Betancor O *et al* (2018) Multiple system atrophy and apolipoprotein E. Mov Disord 33:647–650. 29442376 10.1002/mds.27297 PMC5889322

[29] Orme T , Guerreiro R , Bras J (2018) The genetics of dementia with Lewy bodies: current understanding and future directions. Curr Neurol Neurosci Rep 18:67. 30097731 10.1007/s11910-018-0874-y PMC6097049

[30] Ozawa T , Paviour D , Quinn NP , Josephs KA , Sangha H , Kilford L *et al* (2004) The spectrum of pathological involvement of the striatonigral and olivopontocerebellar systems in multiple system atrophy: clinicopathological correlations. Brain 127(Pt 12):2657–2671. 15509623 10.1093/brain/awh303

[31] Postuma RB , Berg D , Stern M , Poewe W , Olanow CW , Oertel W *et al* (2015) MDS clinical diagnostic criteria for Parkinson's disease. Mov Disord 30:1591–1601. 26474316 10.1002/mds.26424

[32] Rivero‐Gutierrez B , Anzola A , Martinez‐Augustin O , de Medina FS (2014) Stain‐free detection as loading control alternative to Ponceau and housekeeping protein immunodetection in Western blotting. Anal Biochem 467:1–3. 25193447 10.1016/j.ab.2014.08.027

[33] Spillantini MG , Goedert M (2000) The alpha‐synucleinopathies: Parkinson's disease, dementia with Lewy bodies, and multiple system atrophy. Ann N Y Acad Sci 920:16–27. 11193145 10.1111/j.1749-6632.2000.tb06900.x

[34] Stankovic I , Krismer F , Jesic A , Antonini A , Benke T , Brown RG *et al* (2014) Cognitive impairment in multiple system atrophy: a position statement by the neuropsychology task force of the MDS multiple system atrophy (MODIMSA) study group. Mov Disord 29:857–867. 24753321 10.1002/mds.25880 PMC4175376

[35] Thal DR , Rub U , Orantes M , Braak H (2002) Phases of A beta‐deposition in the human brain and its relevance for the development of AD. Neurology 58:1791–1800. 12084879 10.1212/wnl.58.12.1791

[36] Trojanowski JQ , Revesz T (2007) Proposed neuropathological criteria for the post mortem diagnosis of multiple system atrophy. Neuropathol Appl Neurobiol 33:615–620. 17990994 10.1111/j.1365-2990.2007.00907.x

[37] Tsuang D , Leverenz JB , Lopez OL , Hamilton RL , Bennett DA , Schneider JA *et al* (2013) APOE epsilon4 increases risk for dementia in pure synucleinopathies. JAMA Neurol 70:223–228. 23407718 10.1001/jamaneurol.2013.600 PMC3580799

[38] Uchikado H , DelleDonne A , Ahmed Z , Dickson DW (2006) Lewy bodies in progressive supranuclear palsy represent an independent disease process. J Neuropathol Exp Neurol 65:387–395. 16691119 10.1097/01.jnen.0000218449.17073.43

[39] Uchikado H , DelleDonne A , Uitti R , Dickson DW (2006) Coexistence of PSP and MSA: a case report and review of the literature. Acta Neuropathol 111:186–192. 16456665 10.1007/s00401-005-0022-z

[40] Wakabayashi K , Hayashi S , Yoshimoto M , Kudo H , Takahashi H (2000) NACP/alpha‐synuclein‐positive filamentous inclusions in astrocytes and oligodendrocytes of Parkinson's disease brains. Acta Neuropathol 99:14–20. 10651022 10.1007/pl00007400

[41] Wenning GK , Ben‐Shlomo Y , Magalhaes M , Daniel SE , Quinn NP (1995) Clinicopathological study of 35 cases of multiple system atrophy. J Neurol Neurosurg Psychiatry 58:160–166. 7876845 10.1136/jnnp.58.2.160 PMC1073311

[42] Wenning GK , Quinn N , Magalhaes M , Mathias C , Daniel SE (1994) “Minimal change” multiple system atrophy. Mov Disord 9:161–166. 8196676 10.1002/mds.870090206

[43] Wenning GK , Tison F , Ben Shlomo Y , Daniel SE , Quinn NP (1997) Multiple system atrophy: a review of 203 pathologically proven cases. Mov Disord 12:133–147. 9087971 10.1002/mds.870120203

