# Supplementary material for: Clinicopathologic and genetic features of multiple system atrophy with Lewy body disease
Source: Brain Pathol. 2020 Apr 14;30(4):766–78. doi: 10.1111/bpa.12839 (PMC7383746; doi:10.1111/bpa.12839)
Supplement: Supplementary file 1 — Figure S1. Immunoblotting of phosphorylated‐α‐synuclein (upper panels) in MSA (n = 4), MSA+LBD (n = 4), DLBD (n = 4), and Alzheimer's disease (n = 2) using TBS and TBS‐X fractions from the cerebellum (left) and superior temporal gyrus (right). Total protein is shown as loading control (lower panels). Phosphorylated‐α‐synuclein bands are not detected in all samples in TBS and TBS‐X fractions. Table S1. Characteristics of cases for immunoblotting. [file BPA-30-766-s001.doc]

**Supplementary Table 1:** Characteristics of cases for immunoblotting

| Case Number | Pathologic diagnosis | Age | Sex | PMI |
| --- | --- | --- | --- | --- |
| MSA-1 | MSA | 65 | M | 16 |
| MSA-2 | MSA | 68 | F | 10 |
| MSA-3 | MSA | 62 | F | NA |
| MSA-4 | MSA | 70 | F | 4 |
| MSA+LBD-1 (Case 8) | MSA with TLBD | 68 | F | 4 |
| MSA+LBD-2 (Case 9) | MSA with TLBD | 70 | M | NA |
| MSA+LBD-3 (Case 10) | MSA with TLBD | 75 | M | NA |
| MSA+LBD-4 (Case 11) | MSA with DLBD | 67 | M | 4 |
| DLBD-1 | DLBD | 68 | F | 3 |
| DLBD-2 | DLBD | 68 | F | 11 |
| DLBD-3 | DLBD | 70 | M | NA |
| DLBD-4 | DLBD | 63 | M | 11 |
| AD-1 | AD | 73 | M | 4 |
| AD-1 | AD | 65 | F | 7 |

Abbreviation: AD; Alzheimer's disease, DLBD, diffuse Lewy body disease; F, female; M, male; MSA, multiple system atrophy; NA, not available; PMI, Post-mortem interval; TLBD, transitional Lewy body disease.


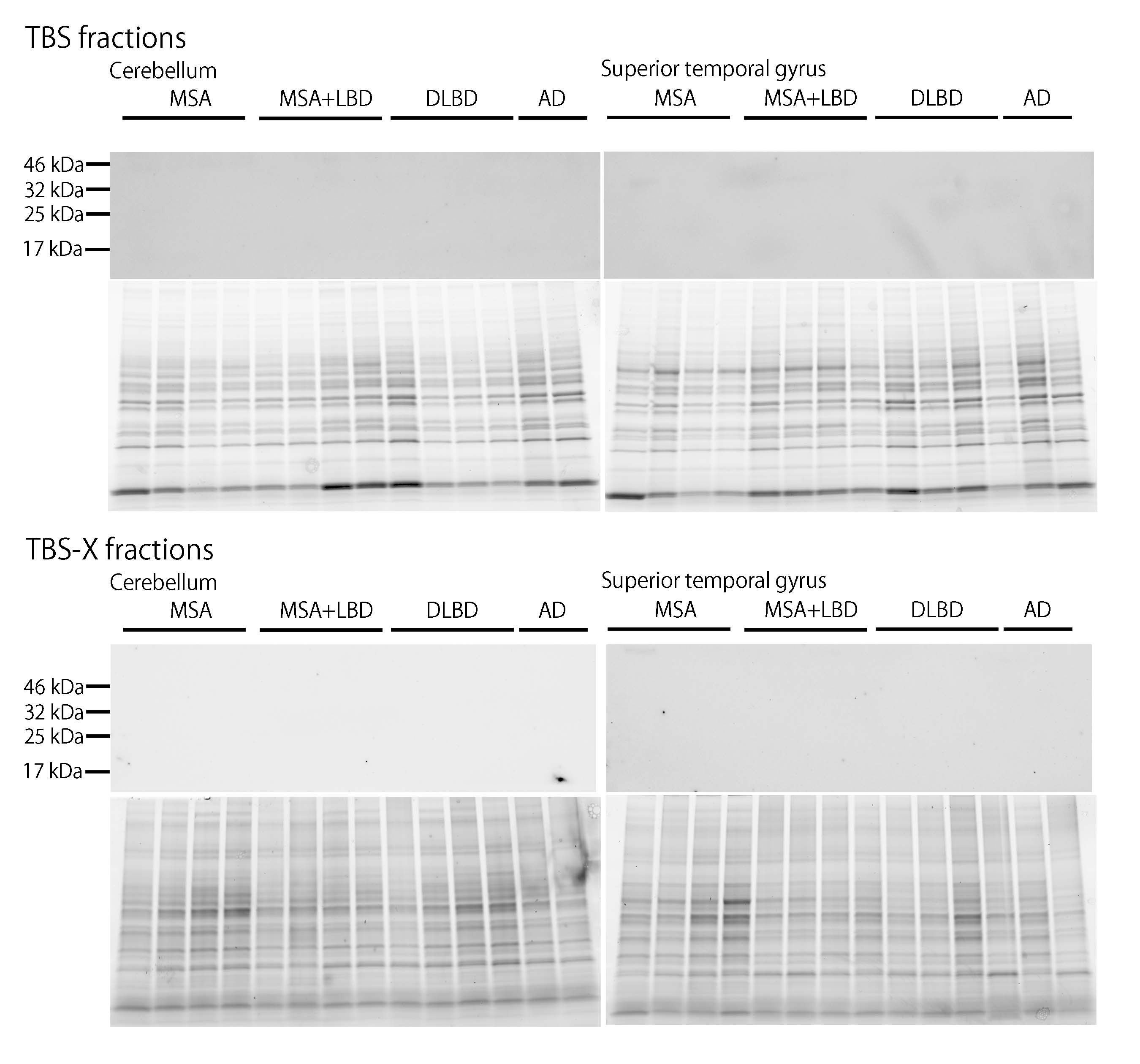


**Supplementary Figure 1**: Immunoblotting of phosphorylated-α-synuclein (upper panels) in MSA (n = 4), MSA+LBD (n = 4), DLBD (n = 4), and Alzheimer's disease (n = 2) using TBS and TBS-X fractions from the cerebellum (left) and superior temporal gyrus (right). Total protein is shown as loading control (lower panels). Phosphorylated-α-synuclein bands are not detected in all samples in TBS and TBS-X fractions.
